# Social Categorisation and Social Identification: The Mediating Role of Social Isolation and Loneliness in Adolescents Living with HIV

**DOI:** 10.1007/s12529-023-10205-x

**Published:** 2023-07-31

**Authors:** Nothando Ngwenya, Thandeka Smith, Maryam Shahmanesh, Christina Psaros, Chiedza Munikwa, Khethokuhle Nkosi, Janet Seeley

**Affiliations:** 1 Africa Health Research Institute, KwaZulu-Natal, Durban, South Africa; 2 University of KwaZulu-Natal, Durban, South Africa; 3 Institute for Global Health, University College London, London, UK; 4 Department of Psychiatry, Harvard Medical School, Massachusetts General Hospital, Boston, USA; 5 Independent Consultant, Polokwane, South Africa; 6 London School of Hygiene and Tropical Medicine, London, UK

**Keywords:** Social isolation, Loneliness, HIV, Adolescents, Stigma, Giddens’ structuration theory

## Abstract

**Background:**

Social isolation and loneliness are associated with living with a chronic condition particularly where stigma is a factor. Our study aimed to examine the lived experience of adolescents living with HIV in relation to isolation because of their diagnosis and consequences of disclosure. Giddens’ structuration theory was used as an analytic framework to identify the potential mechanisms underlying adolescents living with HIV’s experiences.

**Method:**

Longitudinal in-depth interviews were conducted with 20 adolescents living with HIV aged 15–24 years with each participant taking part in three interviews (total 60) between September 2020 and October 2021. Thematic analysis was performed using Braun and Clarke’s steps for coding and analysing qualitative data and informed by the structuration theory framework.

**Results:**

The findings indicated that adolescents living with HIV have agency and make conscious choices about sharing their status. However, these choices are influenced by their experiences in their community. The discrimination and negative judgements they often experience prevent them from disclosing their status. Stigma, discrimination, and psychological distress contribute to the isolation that adolescents and young adults living with HIV experience. The limited disclosure itself can lead to them becoming isolated and lonely.

**Conclusion:**

The negative experiences which adolescents living with HIV face can have an impact not only on their psychological wellbeing but also on their decision to disclose and seek support. These experiences may lead to social isolation and loneliness, an unintended consequence of their action in protecting themselves from the conditions created by the structures/environment in which they live.

## Introduction

In 2020, of the 1.75 million adolescents aged 10–19 living with HIV, 88% lived in sub-Saharan Africa with 360,000 living in South Africa [1]. The availability and scale-up of antiretroviral treatment have changed the face of HIV from a life-threatening to a chronic condition. However, this has not changed the often-distressing psychological impact of HIV infection, especially on adolescents and young adults. HIV is still one of the leading causes of death in sub-Saharan Africa (SSA) and in South Africa particularly accounting for 320,000 deaths in adolescents in 2021 [2]. A young person diagnosed with HIV infection may face many challenges due to long-term antiretroviral exposure, maintaining adherence to their treatment, and frequently experiencing stigma and isolation [3, 4].

HIV is a long-term condition that may require lifestyle adjustments to taking treatment for the rest of the individual’s life [5, 6]. This requires comprehensive clinical services and support to promote effective management of the condition throughout life. As a chronic condition, HIV may affect not only the individual but also their family members, friends, and communities that they interact with [7]. The diagnosis or knowledge of one’s status is often described as being psychologically challenging, traumatic, and acute. Quality of care for people with chronic conditions in resource-limited settings is usually sub-optimal due to a lack of resources. Integrated care models with a patient-centred focus are a potential solution in addressing this. This focus involves the person living with the condition being a partner in their care and taking responsibility for their self-management within the home [7, 8].

Going through adolescence can be challenging [9, 10], and living with a chronic condition like HIV can be disruptive to a young person’s normal life, potentially increasing their risk of developing mental health problems [11, 12]. Adolescence is a transitionary stage between childhood and adulthood where biological and psychosocial changes occur with adolescents developing autonomy from their parents [13]. Being diagnosed with a chronic condition can suddenly thrust a young person into a position of dependency. They may have similar developmental needs to their peers but also have unique needs because of their condition which can be disconcerting and a threat to their wellbeing [13]. Living with a chronic condition can be challenging and demanding and can lead to social restrictions due to stigma [14, 15]. In people living with HIV, psychological distress has a prevalence of up to 35% in SSA with depression being more common than other mental health disorders [11].

Internal and external stigmas contribute to the fear that people living with HIV experience. This can be a barrier to disclosure and seeking of support from family and friends, leading to disengagement from care. Stigma and discrimination within families and communities contribute to barriers to HIV treatment and care, leading to poor health outcomes [16–19]. Negative social consequences of living with HIV can limit the opportunities adolescents have, and at such a precarious developmental stage, this can impair the development of socialisation skills. Interventions that promote social interaction, reduce stigma, and remove barriers to this social support could improve outcomes for adolescents living with HIV [20].

The purpose of this study was to examine the lived experience of adolescents living with HIV in relation to isolation because of their diagnosis and the consequences for disclosure. Social isolation is the absence of social connections, and loneliness is a subjective emotional feeling of isolation due to a lack of social relationships [21]. Issues of social isolation and loneliness can impact the education of young people living with HIV as they might miss school to attend clinics or experience illness due to opportunistic infections. Keeping their status a secret due to fear of stigma from family members and peers can contribute to their psychological distress, consequently resulting in poor mental health. This increases their vulnerability to HIV infection and negatively impacts their quality of life. For people living with HIV in resource-limited settings such as in the global south, this may have major implications on their physical and mental health as they are not always able to access the necessary support. These issues may lead to substance abuse as a coping mechanism and other risky behaviours. Social rejection and isolation lead to poor health outcomes and in some cases human rights abuse due to a lack of knowledge and limited practice of human rights initiatives.

We use Giddens’ structuration theory to examine how the young people describe loneliness and experience isolation due to their condition [22, 23]. Structuration theory seeks to explain the structure of social action and understands human behaviour to be an active and reflexive process. Within structuration theory, human beings are agents or actors who have reasons, intentions, and motives to their actions (behaviour) in response to the forces or structures (society) that they experience [24]. In this paper, we take a narrative approach to describe the actions that adolescents living with HIV as agents of knowledge take, and the structures that influence/enable or constrain their ability to exercise agency.

## Methods

### Participants

Young people aged 15–24 years were eligible to participate if they were living with HIV, were aware of their status and happy to disclose to the research team, lived within the study sites, and could provide informed consent. A purposive sample of twenty adolescents in uMkhanyakude and eThekwini Districts (study sites) were recruited and interviewed for this study. Some participants, identified from the Africa Health Research Institute Database, had previously registered interest to be contacted for studies that they were eligible for. Other participants were approached at the clinic by the peer researchers where they registered interest. All participants were allocated a pseudonym for the purposes of anonymity and were recruited through community-based organisations and local clinics. They were recruited by peer researchers who were themselves adolescents living with HIV, supported by authors TS and NN.

### Recruitment

The study had to be modified as it was conducted during the COVID-19 pandemic where the team had to adhere to physical and social distancing regulations. The peer researchers engaged with community-based organisations (CBOs) who helped identify adolescents living with HIV. The peer researchers then got in touch with potential participants to explain the study and what participating in it would involve. The peer researchers also approached potential participants at the clinics that they themselves attended and gave them information about the research study. Peer researchers explained that participation involved taking part in up to three in-depth interviews as we took a longitudinal approach which allows for the capturing of evolving processes and the changing dynamics in young people’s lives. Some of the participants were known by the peer researchers from various other activities such as support groups for adolescents living with HIV. Those that expressed an interest were then given up to 48 h to consider whether they wanted to participate or not. Following that, one member of the research team (TS) called participants to clarify any queries they had regarding aspects of the research. TS is a Master’s-qualified anthropologist with a background in social health sciences and previous research experience in health systems research. This was then followed by another call with the peer researcher through the Microsoft Teams (MS Teams) platform for a detailed informed consent process which was audio-recorded according to the ethics approvals. The project received permission for parental waiver, and therefore, the participants that were under the age of 18 years (minors) did not require parental consent to participate. The idea to seek parental waiver was from previous studies with young people and from the youth advisory board who expressed concerns that some young people may not have disclosed their status to their parents, and therefore, they would either refuse to take part or the researchers may inadvertently disclose to their parents. Parental waiver was discussed with the Community Advisory Board who gave permission and encouraged maintaining the confidentiality of research participants.

### Ethical Considerations

The study was reviewed and approved by an ethics committee (Ref No: BE321/19). All participants received adequate information about the study, had the opportunity to ask questions and have their questions answered to their satisfaction, and provided informed consent. A form of process consent was used where the peer researchers checked understanding and willingness to participate at each of the three interviews. Parental waiver was approved by the ethics committee and by the community advisory board. Participants were given assurances regarding the confidentiality and anonymity of their information and were informed that they were able to withdraw from the study at any time.

### Data Collection and Analysis

Interviews were conducted in IsiZulu, the local language of the province of KwaZulu-Natal, where this study was conducted between September 2020 and October 2021. They were conducted by the peer researchers with a member of the research team (TS) facilitating as part of the process of supporting the peers. Participants were asked to be in a private space by themselves during the interview, unless they desired for someone else to be in the room. Due to the COVID-19 pandemic, the interviews were conducted via a Voice over Internet Protocol (VoIP) using the MS Teams platform on a tablet that each peer researcher had; thus, we could not ascertain if the participant was by themselves or not. A total of 60 in-depth interviews (IDIs) were conducted as each participant took part in three interviews over the year. Interviews ranged from 15 min to 1 h in length. The data were audio-recorded with permission, transcribed verbatim using a professional transcription service, translated to English, and managed in NVivo 12. The interview topic guide focused on perceptions of health literacy, knowledge of their HIV status, information they received regarding HIV formally and informally, and how the information impacted their everyday decisions. In summary, we asked about young people’s understanding of their diagnosis, its impact on their lives, and their understanding of agency. We also asked questions on decisions they have had to make since receiving their diagnosis, capacity to make those decisions, factors that have negatively or positively influenced their decision-making, consequences of their decisions, and decision-making at school, at home, and in different relationships as well as attitudes and beliefs around HIV-related health literacy. Braun and Clarke’s steps for coding and analysing qualitative data were followed in this analysis process [25]. The first stage of analysis involved working with the peer researchers in broad coding by hand to assist with data familiarisation and interpretation. This was followed by an inductive process of constructing categories through narrative and thematic analysis. Coding was led by NN, TS, and CM and reviewed for quality, internal homogeneity, and external heterogeneity by the other authors [25]. Themes were deductive (theory driven) and inductive (data driven) based on a constant comparison approach using Gidden’s structural theory as a framework. Data saturation was assessed during the constant comparison process as no new themes emerged. Analytical memos informed by the field notes taken after the interviews were used to document findings from emerging themes and interrogate the data in relation to concepts of structuration theory [23].

## Results

Twenty adolescents living with HIV aged between 15 and 24 years took part in three interviews. Fourteen of the participants were female. Of the twenty, ten participants disclosed that they were perinatally infected, seven said they were infected by a partner, and three did not disclose this information. All twenty participants were on antiretroviral therapy (ART) at the time of the interviews. Three participants identified as coloured (mixed race) spoke English, and seventeen were black African with sixteen who spoke IsiZulu and one who spoke SeSotho and was interviewed in English.

We discuss the findings using three overarching themes derived from the data: (i) *Knowledgeable agent — Disclosing HIV status and perceived discrimination* (internalised stigma), (ii) *Social structures — Loneliness and social isolation*, (iii) *Contextuality of social space — Navigating relationships (family, friends, romantic partners, and health care provider)*. We relate those themes to Gidden’s structuration theory to provide an understanding of the lived experience of adolescents living with HIV in relation to isolation because of their diagnosis and the consequences for disclosure. Appropriate direct quotes have been used to illustrate the results in the words of the participants where relevant and appropriate.

**Fig. 1:**
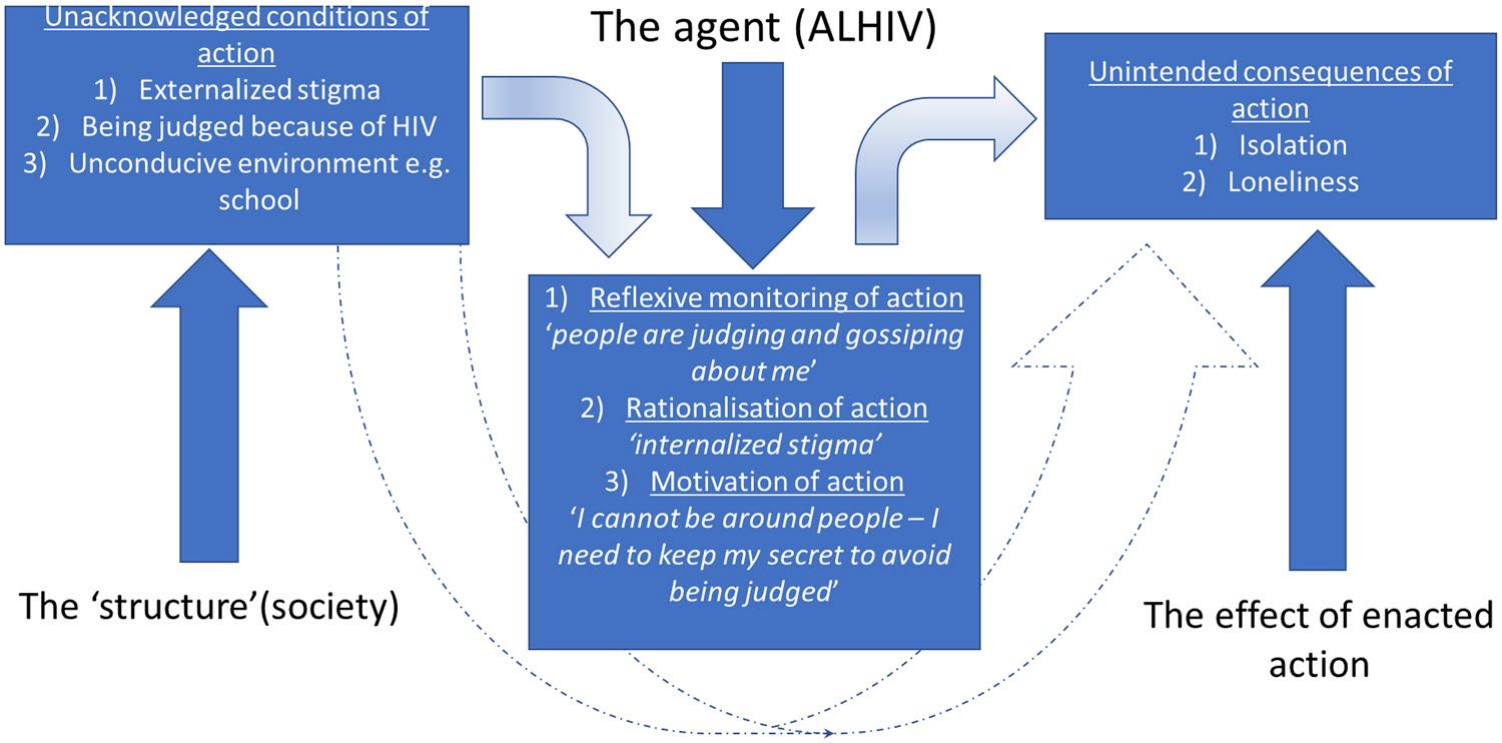
Graphical representation of structuration theory as applied to experiences of adolescents living with HIV in relation to social isolation and loneliness

Figure 1 illustrates the experiences of adolescents living with HIV and how acknowledged conditions of actions by society impact on their actions. These actions lead to unintended consequences of social isolation and loneliness as will be expounded in the “Results” section.

### Knowledgeable Agent — Disclosing HIV Status and Perceived Discrimination

The first theme is related to how adolescents living with HIV had a sense of belief that they knew more about themselves as someone living with HIV than others did and should therefore have the agency to act in relation to when and to whom they can disclose their status. They were aware of the consequences of their action. With this awareness of their agency, they shared how they should be listened to as young people and not be treated as children who have nothing to say. One 23-year-old young woman said:

I’ve never liked getting counselled and stuff, I never liked social workers … [the] reason being, I’ve always been taken to adults, listen to adults, and kids have no say, kids must just listen and not say anything at a younger age.

This theme of self-awareness and agency was also expressed in relation to knowing what to do in relation to disclosing to other people, especially for those in intimate relationships, as one 23-year-old male said:

So, I knew that I was positive, I knew what I needed to do when I was a player, so I was well educated on how to protect myself and how to protect the person that I’m seeing and there had been two people, or should I say three people that found out about my status by me confirming (23-year-old male)

A young man shared that although he was fearful to be with his partner as he is living with HIV, this motivated him to disclose as he said:

I told him after 2 months we met because I was scared to be with him while I know he can get HIV.

The young people portrayed themselves as having the capacity to decide about disclosing their status. They were, however, aware of the consequences of disclosure due to the environmental structures around them which made them make the choice not to share, as a 24-year-old young woman said:

The biggest decision is that I can’t tell people that I am HIV positive. I concluded my decision by not showing people who really I am, … So, I just let them talk, but I do not tell them who really, who am I.

Due to the patterns of practice and the resources adolescents living with HIV have access to, they sometimes may not be able to exercise their agency in disclosing because of their fear of perceived discrimination. One 15-year-old young woman commented:

I have never tried to reveal my status, what if I disclose my status and my peers discriminate against me?

The experience of living with HIV led to these young people fearing rejection by friends and peers and fear of being judged within their community. Their fear seems to come from their experiences and from observation of other people’s experiences, which led to a decision not to disclose their status, as described by a 22-year-old young woman:

they will start judging you and they just bring some negativity to you. I’ve heard some people who had it and they told others, and then they start treating them differently like they can even get it from handshakes, or if you use the same plate.

### Social Structures — Loneliness and Social Isolation

Some participants shared how they struggled to come to terms with knowing their status. In exercising their agency and control in taking the decision not to disclose their status, they harboured a fear of transmitting the virus, which led to the loss of friends, as a 22-year-old young woman said:

I felt a bit broken…I felt I was different from my friends, I was scared to be with my friends, and I started playing alone because I thought I would also affect my friends who do not have it, so it really affected me [be]cause I lost some friends. I never used to play [be]cause I was scared.

They shared how they resorted to social withdrawal to avoid being hurt by other people’s words and actions. Participants felt that being around people was difficult because of the gossip which goes on about people living with HIV, given people sometimes share misinformation about people with the condition, as shared by a 15-year-old girl:

It’s hard for you to be around those people who are saying HIV this and that, so it’s hard…because they are too judgmental. They judge you and then they do not want to hear your side of your story. They think HIV people are, they are so thin, you know, shapeless, ugly I guess, but yeah.

To protect themselves from stigma and judgement by the community and peers, participants resorted to self-isolation. They were worried that they may accidentally disclose their status during social activities and therefore felt safer to be by themselves instead. The feelings of shame through internalised stigma caused emotional turmoil and psychological distress. The psychological distress led to reduced self-worth and suicidal ideation as described by one young man and young woman:

I thought it would be better if I kill myself once I found out that I have it, I was scared to live with it. I felt like my life was finished… when the time comes to drink my medication its painful. (19-year-old male)

I decided to commit suicide by taking an overdose of pills. I then realised that this was a wrong thing to do. I did get help and that is in the past now. (18-year-old female)

### Contextuality of Social Space — Navigating Relationships

Adolescence is a stage when young people are building relationships including beginning to have intimate partners. This was challenging to navigate for some of the participants. They talked about wanting to be “normal” (not be living with HIV) and have what other “normal” young people have, such as a loving relationship and marriage. However, due to their condition, they felt that this desire would not be fulfilled as a 24-year-old woman said:

When it comes to dating. I don’t think I was created for it, although I am a loving person. I am also looking for a partner and I would love to get married someday and just be normal like other people.

Participants talked about how finding a partner was the “norm” and referred to other people (those presumably without HIV infection) as being normal which implied that they did not see themselves as normal.

Some experienced negative reactions and found it difficult to find and maintain relationships, as one 21-year-old young woman said:

…. the one [reaction] that was negative, is when I told my boyfriend I was positive, and then he judged me.

Another young woman (18 years old), on the other hand, described the positive reaction she had on disclosing her status to her boyfriend:

I thought we were going to break up after disclosing my status to him, but he was so understanding and he said there is no problem, we will continue with our relationship.

When navigating the broader social space, young people continue to experience discrimination and judgement which makes them shy away from the wider community.

Even within the school setting, participants did not feel supported or that being in school offered a conducive safe environment. They talked about how people living with HIV were ridiculed at school and how this made them quite uncomfortable as they were aware that should their status be known, they too would be made fun of, as described by a 15-year-old young woman:

At school, there would be lessons about HIV and AIDS and often my classmate would make fun of people living with it. I used to feel out of place and for that they would make me a [the] laughingstock too if they were to find out that I am living with HIV.

They feel unable to discuss their HIV issues, and sometimes, this can be related to sexual health. Some young people shared how sometimes at the clinic they are asked about sexual relations which may need trusting relationships with their care providers to facilitate them getting the support they may need.

## Discussion

In this present study, designed to examine the lived experience of adolescents and young adults living with HIV in relation to isolation and loneliness, findings suggest that young people exercised agency in disclosure of their status. However, due to issues of structural discrimination and fear of stigma, their agency was somewhat curtailed leaving them isolated, lonely, and without social support. The constant fear led to internalised stigma and exacerbated psychological stress.

In reviewing the literature, other studies have reported how young people living with HIV experience challenges related to their developmental stage and the harsh realities of how society responds to HIV as a medical condition [1, 2, 20, 26]. Our results indicate that adolescents living with HIV still experience stigma within their communities. On the aspect of disclosure of HIV status, parents and guardians often do not tell young people that they are living with HIV, and some participants shared that it came as a shock and was somewhat traumatic when they were informed by health care professionals. This was similar to other projects in South Africa, where parents delayed disclosure and so participants were often told by a secondary carer such as a grandparent or extended family such as an aunt [27–29]. This in a way perpetuates the line of thinking that HIV infection is something to be ashamed of, which led to adolescents living with HIV wanting to keep this information a secret. The results of our study showed how living with HIV can have a negative impact on young people’s mental health [19, 30].

As agents, adolescents living with HIV experienced three main aspects which can be linked to the agency and agency proposition concept of structuration theory. The first aspect is the reflexive monitoring of activity (see Fig. 1). The actions and decisions of adolescents living with HIV may be influenced by what they see in their community: other people living with HIV being discriminated. Due to these experiences, adolescents living with HIV decide not to disclose their status in fear of being discriminated and stigmatised. This inadvertently leads to social isolation as they do not want to mistakenly expose their secret.

The second concept of rationalisation of action (see Fig. 1) results in internalised stigma from the experiences of stigma in the community [11]. The constant observations and hearing how people in the community talk about people living with HIV have led adolescents living with HIV to internalise negative stereotypes. They shared how they withhold information about themselves to the extent of having a dual identity, the person they are to themselves and the person they are to others, as they anticipate negative attitudes and discrimination [31]. In their motivation to action (see Fig. 1), adolescents living with HIV lost some relationships or found them difficult to maintain as they felt like misfits and as they described or referred to others (people without HIV infection) as “normal”, implying a notion of them not being “normal”. They at times pulled away from other friendships due to their condition. Our findings are consistent with previous research that has shown how people living with HIV experience social isolation due to their chronic condition [26, 32]. There is also a useful comparison between these results and those found with other chronic diseases that showed the positive association of social isolation and loneliness to a diagnosis of cardiovascular diseases and diabetes mellitus type 2 [33].

The effects of social isolation and loneliness are detrimental especially in adolescence where it can have a lasting impact. Humans are social beings and more so in adolescence where young people are developing the skills to interact with others on an individual basis. Not only can isolation lead to psychological distress and negative aspects such as bullying, being alone can disrupt brain development, lead to impaired executive function and cognitive decline, and can impact/change decision-making in adulthood [34, 35].

## Limitations

We were unable to do comparative analysis between urban and rural participants which would have added more on context- and setting-based nuances that can contribute to targeted intervention development. Data from the male participants was quite sparse as they were not all very engaged in terms of sharing about their experiences, even though four of them were interviewed by a male peer researcher. A potential explanation could be issues of gendered power through masculinity ideals where young boys do not publicly share or verbalise their concerns, experiences, and anxieties; however, this was not explored within this study.

## Conclusions

Our theoretical approach to social isolation and loneliness provided a way of identifying the mechanisms of social isolation and loneliness among young people living with HIV by highlighting the social structures and the context that may inhibit these young people from having agentic capacity. This approach also highlights how social isolation and loneliness are malleable behaviours and therefore could be targeted through interventions that would support adolescents living with HIV. Although consciously they may want to change their situation, the risks of disclosing were too high for most of them.

Our findings highlight how HIV remains a stigmatised condition that leaves young people socially isolated and in a vulnerable position of loneliness. With the high magnitude of risk of social isolation and loneliness and long-term impacts, it is essential that this aspect of growing up with HIV is addressed. Developing effective interventions can be challenging as there are many sources of loneliness; however, using theory to identify potential mechanisms and pathways can be a useful approach. To this end, structuration theory helps to clarify that combination interventions are necessary to target the broader social structures (addressing social stigma) and the individual’s self-esteem, stimulating their agentic capacity.

## Data Availability

The data that support the findings of this study are available but restrictions apply due to confidentiality reasons, and so are not publicly available. The data are, however, available from the authors upon reasonable request to the corresponding author.

## Declarations

### Statement Regarding the Welfare of Animals

This article does not contain any studies with animals performed by any of the authors.
